# FA-Net: A Fused Feature for Multi-Head Attention Recoding Network for Pear Leaf Nutritional Deficiency Diagnosis with Visual RGB-Image Depth and Shallow Features

**DOI:** 10.3390/s23094507

**Published:** 2023-05-05

**Authors:** Yi Song, Li Liu, Yuan Rao, Xiaodan Zhang, Xiu Jin

**Affiliations:** 1 College of Information and Computer Science, Anhui Agricultural University, Hefei 230001, China; 2 College of Horticulture, Anhui Agricultural University, Hefei 230001, China

**Keywords:** nutrient deficiencies, deep learning, feature fusion, convolutional neural networks, attention mechanism

## Abstract

Accurate diagnosis of pear tree nutrient deficiency symptoms is vital for the timely adoption of fertilization and treatment. This study proposes a novel method on the fused feature multi-head attention recording network with image depth and shallow feature fusion for diagnosing nutrient deficiency symptoms in pear leaves. First, the shallow features of nutrient-deficient pear leaf images are extracted using manual feature extraction methods, and the depth features are extracted by the deep network model. Second, the shallow features are fused with the depth features using serial fusion. In addition, the fused features are trained using three classification algorithms, F-Net, FC-Net, and FA-Net, proposed in this paper. Finally, we compare the performance of single feature-based and fusion feature-based identification algorithms in the nutrient-deficient pear leaf diagnostic task. The best classification performance is achieved by fusing the depth features output from the ConvNeXt-Base deep network model with shallow features using the proposed FA-Net network, which improved the average accuracy by 15.34 and 10.19 percentage points, respectively, compared with the original ConvNeXt-Base model and the shallow feature-based recognition model. The result can accurately recognize pear leaf deficiency images by providing a theoretical foundation for identifying plant nutrient-deficient leaves.

## 1. Introduction

Pear is the world’s third-largest fruit tree industry, and pear tree fertilizer demand is high, so blind fertilization easily appears as soil barriers and a lack of nutrients. When the pear tree is deficient in nutrients, the appearance of the color and texture of the leaves will show the related symptoms of nutrient deficiency [1]. Accurately diagnosing nutrient deficiencies in plant leaves is a crucial process for enhancing agricultural production and improving crop quality and yield. Experienced staff in the field usually identify plant leaf nutrient deficiencies visually, but this is very subjective and time-consuming.

In recent years, some studies have yielded many results using image-processing techniques for identifying nutrient deficiency in plants [2,3]. Hossain E. et al. [4] proposed a technique for plant leaf disease detection and classification using a K-nearest neighbor (KNN) classifier. The texture features are extracted from the leaf disease images for the classification. Devechio F.F.S. et al. [5] proposed an image processing method with texture analysis to identify maize’s calcium (Ca) deficiency using an artificial vision system (AVS). Anami B.S. et al. [6] used the color moment feature, the primary color feature, and the color layout feature methods for feature extraction, then the SFFS method for feature selection, and finally, BPNN, SVM, and k-NN classifiers were used to classify nutrient-deficient crops. Lisu C. et al. [7] used the R, G, and B mean functions and the area props function to extract the color and shape characteristic parameters of leaves and leaf tips and then used SVM to diagnose the nutrient deficiency status of rice. Sabri N. et al. [8] extracted the gray-level co-occurrence matrix (GLCM) and color histogram feature parameters of maize leaves, used the Random Forest as a classifier, and obtained 78.35% accuracy. Considering the above study, the traditional plant nutrient deficiency diagnosis method mainly extracts the color, shape, texture, chlorophyll distribution, and other features of nutrient-deficient leaves through preprocessing image technology, then carries out species identification through a machine learning model [9,10,11,12,13].

Deep learning is widely used to identify plant nutrient deficiencies and insect diseases [14,15,16,17,18,19]. Tran et al. [20] used two prediction models based on CNN structure to achieve the prediction and classification of tomato leaves and fruit nutrient deficiency symptoms, and the accuracy of deficiency identification of models Inception-resnet v2 and Autoencoder increased from 87.27% and 79.09% to 91%. Yi et al. [21] proposed a new sugar beet nutrient deficiency dataset and used it as a benchmark to evaluate the performance of five convolutional neural networks to identify nutrient deficiency symptoms. Although deep learning methods have been shown to be very capable of extracting image depth features, in the absence of a large amount of labeled training data, they still face the problem of inadequate feature extraction. Therefore, Fan et al. [22] used feature fusion to combine depth features with traditional manual features to obtain local texture information from plant leaf images and conducted extensive experiments on three publicly available datasets. The experimental results showed that the feature fusion approach effectively obtains discriminative feature representations of plant leaf diseases. Zhang et al. [23] designed a multi-feature fusion Faster R-CNN (MF3 R-CNN) to detect soybean leaf diseases in complex scenarios and obtained an optimal average accuracy of 83.34% in a real test dataset. The study mentioned above demonstrates that feature fusion techniques can be an effective approach to enhancing model classification performance.

Most of the current research with plant images has focused on the identification of plant species and pathology, with less application in the field of plant nutrient deficiency identification. To achieve an efficient diagnosis of plant nutrient deficiencies, it is crucial to extract effective features from the images [24]. Given that pear leaf nutrient deficiency often leads to changes in leaf color and texture, it is possible to diagnose this deficiency by extracting color and texture features from images of pear leaves. Within color features, HSI channels are widely used because they can effectively express the differences between nutrient-deficient and healthy leaves [25,26]. In texture features, the gray-level co-occurrence matrix (GLSM) can express the combined information of the image regarding orientation, adjacent interval, and magnitude variation [11,27]. Compared with color and texture features, deep convolutional networks and attention mechanisms can greatly extract the depth feature information of images [28,29]. Mi Z. et al. [30] constructed a C-DenseNet network based on the attention mechanism to achieve wheat stripe rust classification under field conditions. The experimental results showed that the test accuracy of C-DenseNet was 97.99%, which was better than the classical DenseNet (92.53%) and ResNet (73.43%). Zhao et al. [31] constructed a DTL-SE-ResNet50 vegetable disease identification method based on attention mechanisms and transfer learning methods and discussed the effect of the attention mechanism on model performance. The results show that the model has high accuracy in real-life scenarios. Manually extracted shallow features are beneficial for understanding and interpretation, but their representational power is limited. Depth features learned through neural networks can express higher-level abstract concepts, but these features are difficult to understand and interpret. By fusing depth features learned through neural networks with shallow features manually extracted, we can leverage the strengths of both to create more accurate and robust models.

Therefore, we construct a pear leaf nutrient deficiency image dataset and propose a nutrient-deficient pear leaf diagnosis method based on depth and shallow feature fusion. First, texture features and color features of nutrient-deficient pear leaf images are extracted as shallow features. The ConvNeXt-Base network is used as the feature extractor to output image depth features. Finally, the fused shallow and depth features are trained by the three algorithms F-Net, FC-Net, and FA-Net proposed in this study to achieve accurate identification of nutrient-deficient pear leaves. The article concludes with a summary and discussion of the performance of the diagnosis model for pear leaf nutrient deficiency based on fused features.

## 2. Materials and Methods

### 2.1. Sample Acquisition

The materials for this experiment were obtained on 26 April and 10 May 2021, in the pear orchard of Anhui Agricultural University High-Tech Park, using several common nutrient deficiency diseases of pear leaves: nitrogen (N) deficiency, phosphorus (P) deficiency, potassium (K) deficiency, iron (Fe) deficiency, and magnesium (Mg) deficiency as the study subjects, with healthy leaves as the control. To ensure the quality of the pictures, the collected leaves were free from invasive diseases, insects, and breakage. Pear leaves were collected using a random sampling method to ensure that they were obtained from different aspects of each tree.

**Figure 1:**
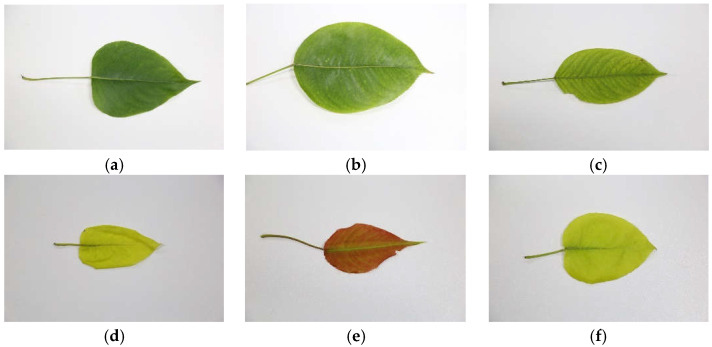
Examples of nutrient-deficient pear leaves. (**a**) Healthy leaf; (**b**) K-deficient leaf; (**c**) Mg-deficient leaf; (**d**) N-deficient leaf; (**e**) P-deficient leaf; and (**f**) Fe-deficient leaf.

The collected samples were taken back to the laboratory in sealed bags and placed in a 4 °C refrigerator for temporary storage, and the experiment included a total of 1314 leaf samples. Before collecting the images, the dust and debris on the leaves were removed with paper towels. The equipment used to take the pictures was a Canon EOS 6D; the resolution of the images was set to 5472 × 3648 pixels; and the ISO speed was ISO-1600. Different nutrient-deficient and healthy pear leaf image samples are shown in Figure 1.

N is a component of amino acids, proteins, nucleic acids, and other substances in plants. In the case of N deficiency, the leaves lose their green color uniformly and are yellowish-green or yellowish. P plays crucial roles in sugar metabolism, protein metabolism, and lipid metabolism in plants. In the case of P deficiency, the leaves are small and thin, dark green, and in severe cases K is involved in respiration and photosynthesis processes and can promote sugar synthesis and increase cellulose and lignin content. When potassium is insufficient, the lower leaves or the middle leaves of the branches lose their greenish-yellowish edges, and the leaves are often crinkled. Mg plays a key role in chlorophyll synthesis and is involved in various plant metabolic processes. When the pear leaf is deficient in Mg, the leaves become yellowish between the veins with yellow-white spots, and the whole leaf is ribbed to lose green. Fe has a vital role in the synthesis of some chlorophyll-protein complexes in plants. Therefore, Fe deficiency causes the leaves to turn yellowish-white, and the whole leaf appears reticulate. Table 1 shows the diagnostic indicators of the elemental content of pear leaves.

### 2.2. Shallow Feature Extraction Method for Visual Images

The background must be eliminated before image analysis, since the image of pear leaves contains many background components. To ensure that the leaves and the background can be effectively separated, the image of pear leaves is segmented by iterative thresholding [33]. First, choose an approximate threshold T, split the image into two parts, $R_{1}$ and $\;R_{2}$, calculate the mean values $u_{1}$ and $\;u_{2}$ of regions $R_{1}\;$ and $\;R_{2}$, then choose a new threshold $\;T=\left(u_{1}+u_{2}\right)$. Repeat the above process until $u_{1\;}$ and $u_{2}\;$ no longer change. The nutrient deficiency of pear leaves is reflected in the leaf color. To scientifically determine, study, and use color, more than a dozen color models have been established, including RGB, HSI, CMYK, XYZ, and other commonly used color description models. In the color feature extraction, this study uses the HSI color model that is commonly used in image processing [34,35]. Hue (H) can be expressed as various color types, such as RGB; intensity (I) is used to indicate the lightness and darkness of the image; and saturation (S) is used to indicate the vividness of the color. The formula for calculating the HSI color system’s H, S, and I is as follows: R , G, and B represent the brightness values of red, green, and blue in the image, respectively.

(1) I=(R+G+B)/3

(2) H=arccosR−G+R−B2R−G2+R−BG−B12

(3) $$S=1-\frac{3}{\left(R+G+B\right)}\left[\min\left(R,G,B\right)\right]$$

The texture is an essential feature of an image and describes the grayscale of the image pixels. The classical algorithm for texture analysis is the gray-level co-occurrence matrix (GLSM) method [36]. It is necessary to convert color images to grayscale images before performing texture analysis on the images of nutrient-deficient pear leaves. The following six most commonly used features are generally used to extract the texture features of the image: The angular second moment (ASM) reflects the uniformity of the image grayscale distribution and the coarseness of the texture; the contrast ratio reflects the sharpness of the image and the depth of the texture grooves; the correlation is used to measure how similar the elements of the grayscale co-occurrence matrix are in the row or column direction; the entropy is used to measure the amount of information in an image; the inverse difference matrix (IDM) reflects the degree of clarity and regularity of the texture; and the homogeneity measure is used to measure the amount of local variation in image texture.

**Figure 2:**
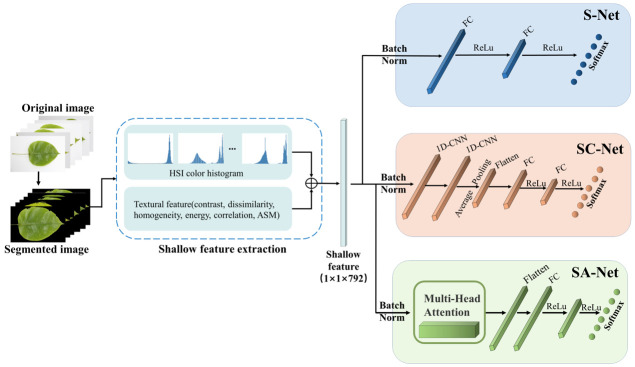
Flow chart of the shallow feature-based identification algorithm.

The specific method for visual image shallow feature extraction is as follows: First, extract the image’s shallow color features using the HSI color model; extract the image’s shallow texture features using the GLCM; then concatenate the color features and texture features to form image shallow features; and finally, use the classification algorithms based on the shallow features proposed in this chapter to classify. The color histogram of the HSI color space is selected as the color feature of the image, and the color histograms of the H, S, and I channels form a 1 × 768-dimensional shallow color feature vector. The six feature parameters of the GLCM are extracted in the 0°, 45°, 90°, and 135° directions to form a 1 × 24-dimensional shallow texture feature vector. The shallow feature-based identification algorithm is shown in Figure 2.

Interconnect the extracted shallow color and texture features to form new shallow feature vectors. Subsequently, they are input to the classification algorithms based on the shallow features of the Shallow Feature Fully Connected Network (S-Net), the Shallow Feature CNN Recoding Network (SC-Net), and the Shallow Feature Multi-Head Attentive Recoding Network (SA-Net) to output the corresponding classification results. The S-Net uses fully connected layers for the classification of the shallow feature vectors. The SC-Net uses one-dimensional convolution (1D-CNN) to recode the shallow feature vector and adds the fully connected layer for classification. The SA-Net uses a multi-head attention module to recode the shallow feature vector and finally uses the fully connected layer for classification.

Using the above three shallow feature-based classification algorithms to classify the extracted shallow features, the validity of the extracted shallow features can be evaluated.

### 2.3. Depth Feature Extraction Method for Visual Images

Neural networks automatically learn the intrinsic and abstract features of the target through a large number of data samples, which are called depth features. Compared with artificially constructed shallow features, depth features do not require human participation and increase the process of model learning, so depth features are generally less affected by external factors such as lighting.

VGG16 is a classical convolutional neural network model proposed by Simonyan et al. [37] in 2014 that is widely used in image classification. The basic structure of this model includes an input layer, a convolutional layer, a pooling layer, a fully connected layer, and an output layer. The convolutional layer uses this convolutional kernel to perform convolutional operations to extract the features of the input image; the pooling layer performs feature dimensionality reduction to reduce the complexity of the network; and the final fully connected layer and the output layer classify the results.

Dosovitskiy et al. [38] first applied the original Transformer model to the image classification task in October 2020, proposing the Vision Transformer (ViT) model, an image classification scheme based entirely on self-attention mechanisms. Each image block is flattened into a one-dimensional vector, and then a linear projection transformation is applied to each vector while a position encoding is raised to include the position information of the sequence. In addition, a classification flag bit is added before the input sequence data to better represent the global information.

In this paper, we first select the ViT-L/16 model in the ViT series that performs well on the ImageNet22K dataset [39]. The ViT-L/16 model is usually pretrained on large datasets and fine-tuned for smaller downstream tasks. ViT-L/16 performs well in classification tasks on large-scale datasets without relying on CNN.

Although ViT-L/16 shows excellent performance in computer vision tasks, it cannot replace convolutional neural networks in image processing at present. Facebook AI Research proposed the ConvNeXt convolutional neural network [40] in 2022. Through a few experimental comparisons, ConvNeXt has better performance than Swin Transformer. Their research shows that CNN networks can still achieve competitive performance in image identification and classification.

**Figure 3:**
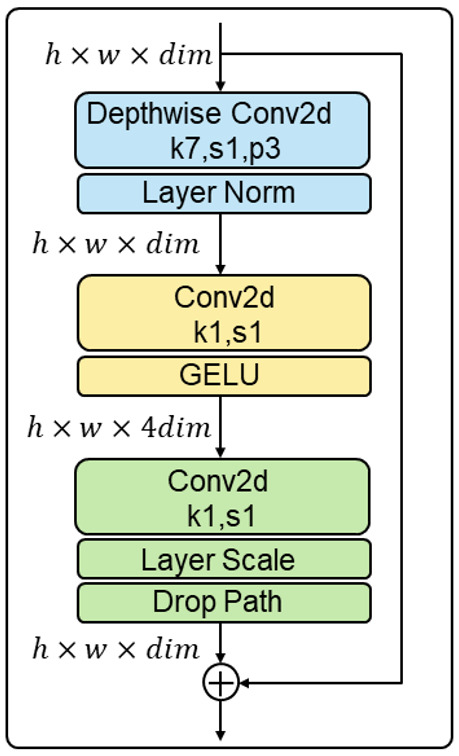
The block of ConvNeXt.

ConvNeXt uses the inverted bottleneck structure to reduce the loss of information, and the block of ConvNeXt is shown in Figure 3. This structure has been tested to partially reduce the parameter size of the model, with a slight increase in accuracy, and improve the model’s overall performance. In classical CNN networks, we usually use 3 × 3 convolutional kernels, but ConvNeXt tests various sizes of convolutional kernels and finds that the accuracy and parameter size of the network are optimal when the size of the convolutional kernels is 7. Traditional CNN networks usually use RELU as the activation function of the network, but the GELU activation function is mainly used in Transformer-type networks. Therefore, the ConvNeXt network tries to replace the RELU activation function with the more commonly used GELU activation function and reduce the number of activation functions so that the performance of the network can be slightly improved. Meanwhile, ConvNeXt uses fewer normalization layers and replaces BatchNorm with LayerNorm.

**Figure 4:**
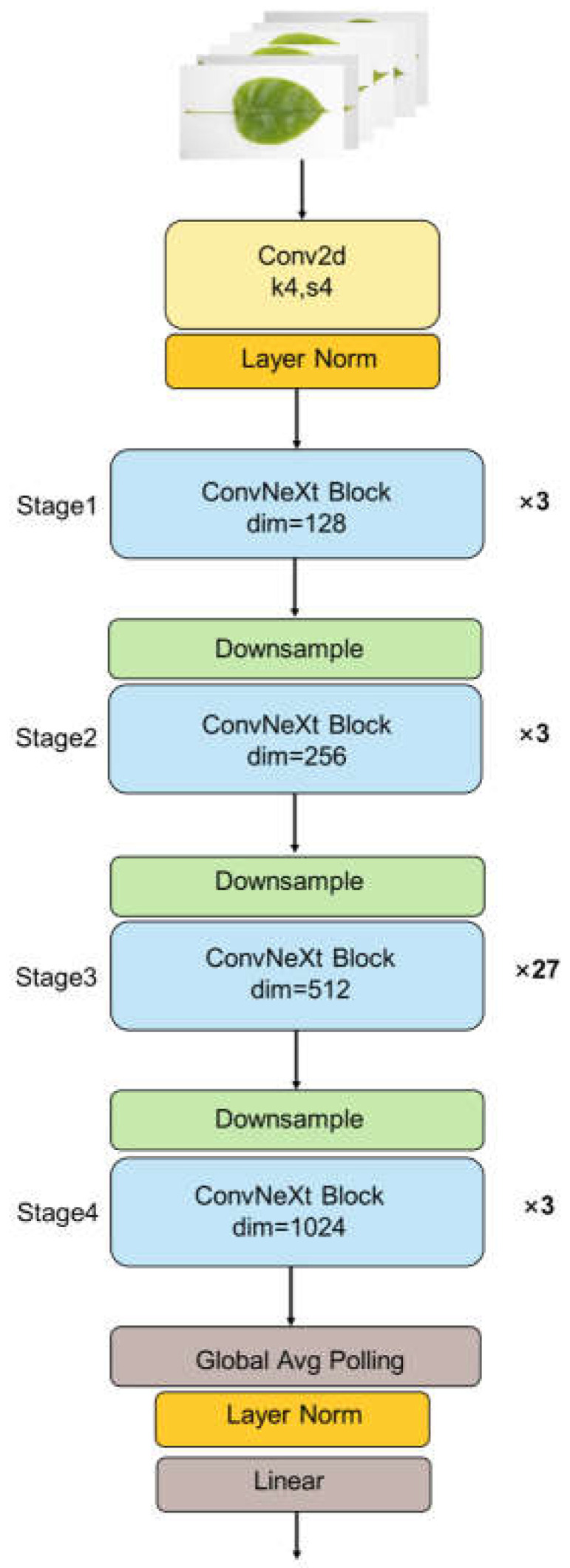
The structure of ConvNeXt-Base.

In this study, we chose the ConvNeXt-Base network version, as Figure 4 shows the structure diagram of the ConvNeXt-Base model (ConvNeXt-Base: C = (128, 256, 512, 1024), B = (3, 3, 27, 3), where C represents the number of input channels in the four stages, and B represents the number of times the blocks are repeatedly stacked for each stage).

**Figure 5:**
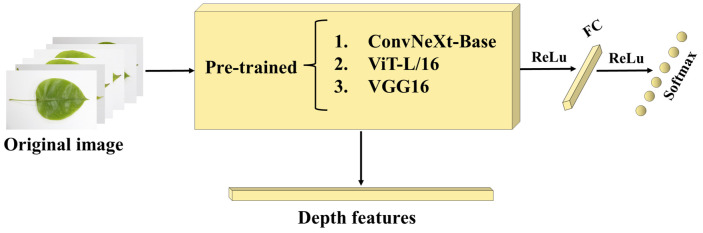
Flow chart of the depth feature-based identification algorithm.

In this study, we fine-tune the network using pre-trained models, and the specific procedure is as follows: We downloaded the pre-training weights of the ConvNeXt-Base, VGG16, and ViT-L/16 on ImageNet22K as the initialization of the network parameters. The network models were then trained using the image dataset of nutrient-deficient pear leaves. The final depth features are obtained from the output of the last normalization layer of the trained deep network model for the further fusion process. The depth feature-based identification algorithm flow is shown in Figure 5.

### 2.4. Depth and Shallow Feature Fusion Method

Pear leaves under different nutrient deficiency conditions may display different color and texture features. To better capture the features, this study proposes a depth and shallow feature fusion method that fuses depth features extracted by deep neural networks with manually extracted shallow features such as texture features and color features. The advantage is that it can combine the depth and shallow features of nutrient-deficient pear leaves to provide more important feature information. Furthermore, we propose three classification algorithms based on fused features: the Fused Feature Fully Connected Network (F-Net), the Fused Feature CNN Recoding Network (FC-Net), and the Fused Feature Multi-Head Attention Recoding Network (FA-Net). Among them, the FA-Net uses multi-head attention to recode the fused features.

**Figure 6:**
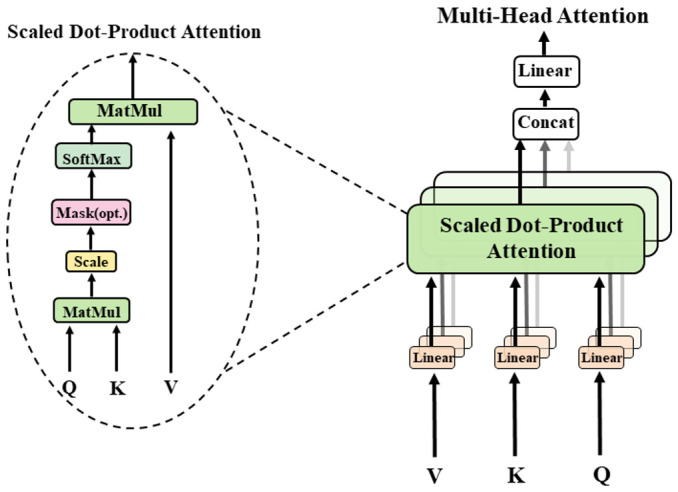
Multi-Head Attention consists of several attention layers running in parallel, which is a redrawn figure of Figure 2 from the cited paper [41].

Google Inc. proposed a multi-head attention mechanism in 2017 that uses scaled dot-product attention [41], as shown in Figure 6. The scaled dot-product attention computes the dot products of the query with all keys, divide each by $\sqrt{D_{k}}\;$, and applies a softmax function to obtain the weights on the values. The corresponding equation is shown in (4). $D_{k}\;$ is the number of columns of the metrices Q and K, i.e., the vector dimension. The query (Q) represents the information that the model needs to search for; the key (K) is the index used to retrieve information; and the value (V) is the actual information itself. The importance of value is determined by calculating the similarity between query and key. Furthermore, the weighted sum of important values is used to obtain the final output of the model. The formula for linear conversion of the input is shown in (5); $X=\left[x_{1},x_{2},x_{3,\cdots \;,}\;x_{n}\right]$ represents input information; and $W_{q},W_{k},W_{v}\;$ are the weight matrices obtained in the training.

(4) $$Attention\left(Q,K,V\right)=softmax(\frac{QK^{T}}{\sqrt{D_{k}}})V$$

(5) $$Q=W_{q}X\;K=W_{k}X\;V=W_{v}X$$

In a multi-head attention mechanism, each set of segmented parameter inputs is projected onto a different subspace of the high-dimensional space. Attention weights are then computed in each subspace using the scaled dot-product attention, which allocates attention to the input features in each subspace separately. Finally, the outputs of each subspace are concatenated together to form the final output. This approach enhances the model’s ability to focus on input information and improves its performance. The formula is shown in (6). $W^{0\;},W_{i}^{Q},\;W_{i}^{K},\mathrm{and}\;W_{i}^{V}$ are the parameter matrices for the linear transformation.

(6) $$MultiHead\left(Q,K,V\right)=Concat\left(head_{1},\ldots ,head_{h}\right)W^{0\;}\;\mathrm{where}\;head_{i}=Attention{\left(QW_{i}^{Q},KW_{i}^{K},VW_{i}^{V}\right)}^{\;}$$

Since attention is distributed differently in different subspaces, multi-head attention looks for associations between different perspectives of the input data so that multiple relationships and nuances can be encoded. Multiple independent heads of the multi-head attention mechanism can focus on different information simultaneously, both global and local, to extract more comprehensive and rich features.

**Figure 7:**
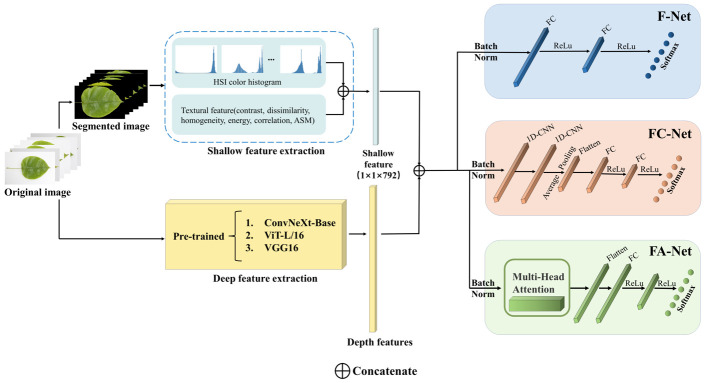
Flow chart of the identification algorithm based on depth feature and shallow feature fusion.

The process of depth and shallow feature fusion and the classification of fused features are shown in Figure 7. Initially, the image’s shallow color and texture features are extracted through the method described in Section 2.2. The resulting features are then combined to create shallow feature vectors. Next, the depth feature extraction method proposed in Section 2.3 is used to extract the image’s depth features. These depth features are combined with the previously extracted shallow features to create new fused feature vectors. In the final step, the newly composed fused feature vectors are fed into the Fused Feature Fully Connected Network (F-Net), the Fused Feature CNN Recoding Network (FC-Net), and the Fused Feature Multi-Head Attention Recoding Network (FA-Net) proposed in this section to output the corresponding classification results. The F-Net uses fully connected layers for the classification of fused feature vectors. The FC-Net uses one-dimensional convolution (1D-CNN) to recode the shallow feature vector and adds the fully connected layer for classification. The FA-Net uses a multi-head attention module to recode the shallow feature vector and finally uses the fully connected layer for classification. Using the ReLu activation function in the neural network can reduce the interdependence between parameters and moderate the overfitting problem. Finally, we compare and analyze the classification effects of the three networks on the fused features.

### 2.5. Evaluation Indicators

True positive (TP) is the number of positive classes predicted to be positive. True negative (TN) is the number of negative classes predicted to be negative. False positive (FP) is the number of negative classes predicted to be positive. False negatives (FN) are the number of positive classes predicted as negative classes. Precision (precision, P, %), which is the proportion of the number of samples predicted to be in the positive category that are actually in the positive category, was calculated as follows:

(7) $$P=\frac{TP}{TP+FP}\times 100\%$$

Recall (recall, R, %), which is the proportion of the sample predicted to be a positive class that is actually a positive class, was calculated as follows:

(8) $$R=\frac{TP}{TP+FN}\times 100\%$$

The F1-score (F1, %) is a combination of precision and recall. When F1 was high, the classification method was effective. The calculation method is as follows:

(9) $$F1=\frac{2\times P\times R}{P+R}\times 100\%$$

Average precision (AP, %) measures the identification accuracy of an algorithm on a dataset, and average precision is defined as follows:

(10) $$AP=\sum_{i=1}^{N}\frac{TP_{i}}{TP_{i}+FP_{i}}$$

The accuracy (accuracy, Acc, %), which is the number of samples correctly classified divided by the total number of samples, was calculated as follows:

(11) $$Acc=\frac{TP+TN}{TP+TN+FP+FN}\times 100\%$$

## 3. Results and Discussion

### 3.1. Sample Analysis

In this study, we collected 1314 images of pear leaves as experimental data, including five types of nutrient-deficient leaves and one type of healthy leaf, and we divided the training set, validation set, and test set according to a ratio of 7:2:1. The results are shown in Table 2.

All models were trained using Python 3.9 and the PyTorch framework, with 16 GB of computer memory, an Intel Core i5-12500H CPU, and an NVIDIA GTX3050Ti GPU to accelerate the deep learning models. The model was optimized using the Adam optimizer with an initial learning rate of 0.001 and further adjusted to 0.0001, with 150 iterations of training for each model, and the number of batches was 8.

### 3.2. Analysis of Depth Feature and Shallow Feature Extraction of Vision Images

**Figure 8:**
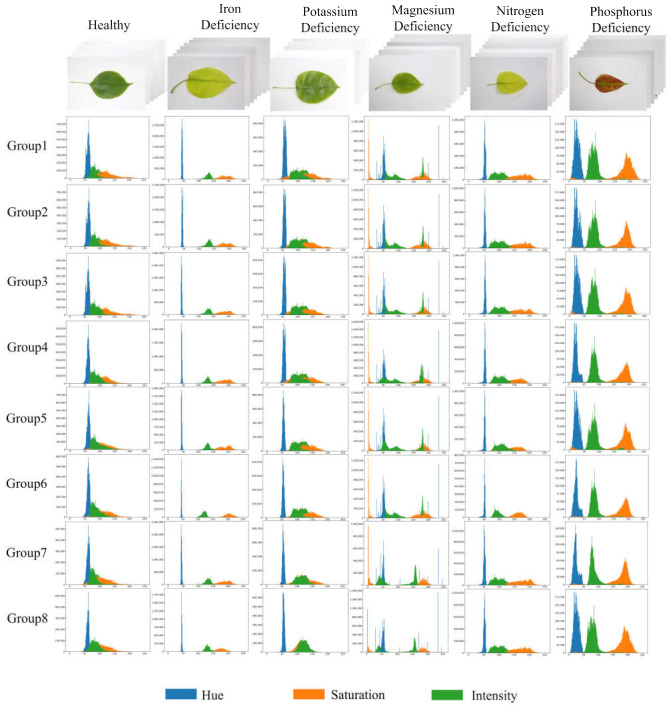
Sample results of the HSI color histogram.

In order to demonstrate the variations in color characteristics among pear leaves affected by different nutrient deficiencies, we randomly selected eight leaves from each deficiency type and created HSI color histograms for each group, resulting in a total of eight control groups. Figure 8 shows the control results of the shallow color features. From the extraction results of the shallow color features, it can be well distinguished between Fe-deficient leaves, Mg-deficient leaves, N-deficient leaves, and P-deficient leaves by the feature information of the HSI color histogram of the three channels H, S, and I. The color histogram features of healthy and K-deficient leaves are similar, and it is difficult to distinguish them by the color features alone.

**Figure 9:**
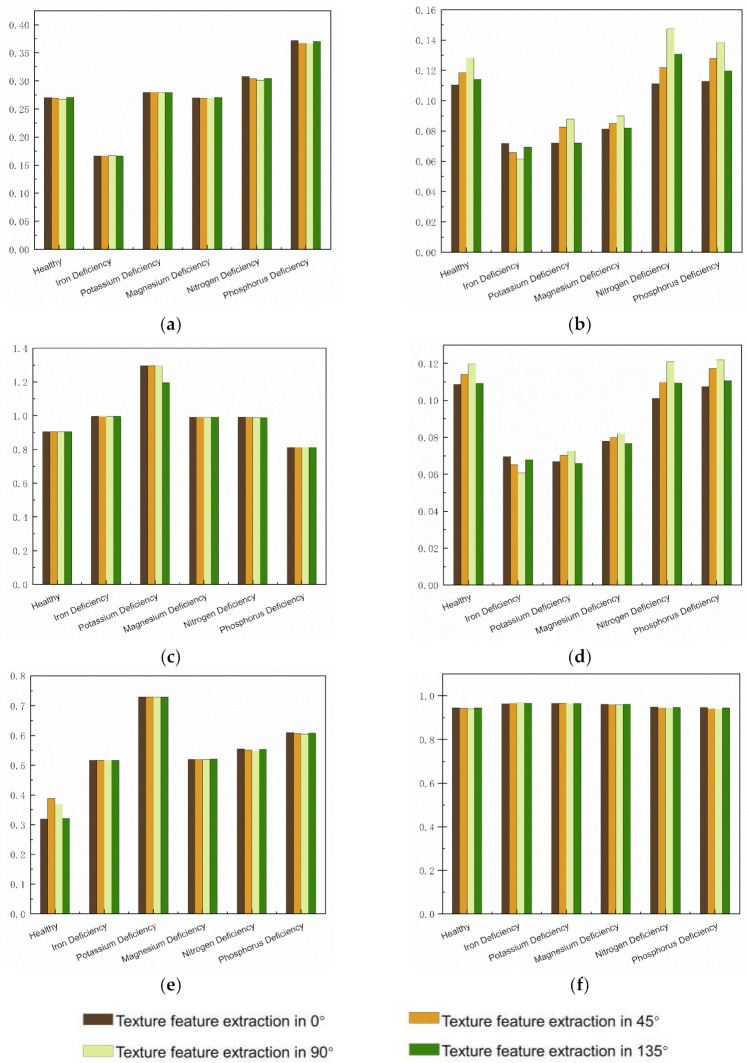
Sample result of GLSM texture feature extraction. (**a**) ASM; (**b**) contrast; (**c**) correlation; (**d**) DISL; (**e**) energy; and (**f**) homogeneity.

Figure 9 shows the extraction results of the shallow texture features. From the extraction results of the shallow texture features, the four parameters of the GLCM, namely, contrast, correlation, DISL, and energy, can be used to distinguish healthy leaves from potassium-deficient leaves. The GLCM features compensate for the lack of light color features, but relying on the GLCM feature parameters alone makes it difficult to distinguish other types of pear leaves.

**Figure 10:**
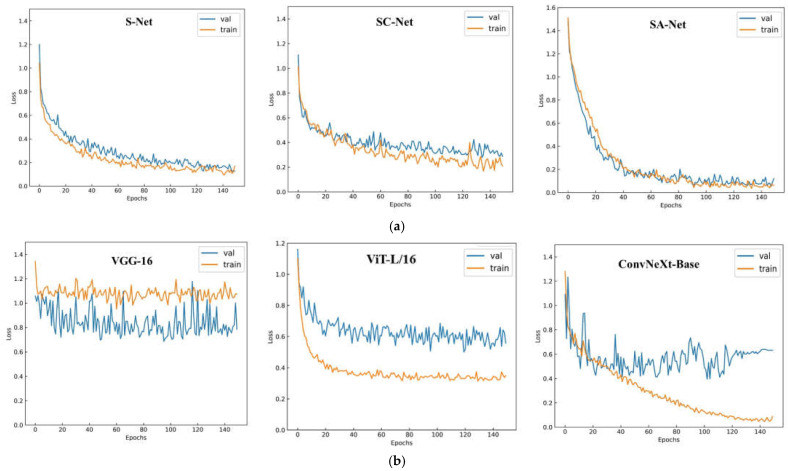
(**a**) Loss variation curve of the shallow feature-based identification algorithms. (**b**) Loss variation curve of the depth feature-based identification algorithms.

Figure 10a,b show the loss variation curves of the shallow feature-based identification algorithms and the depth feature-based identification algorithms, respectively, and Table 3 shows the average accuracy of the two types of algorithms on the test set. It can be seen that all three shallow feature-based identification algorithms can obtain low loss values. However, the highest accuracy of the algorithm on the test set was only 84.6%, indicating that the shallow features cannot be fully utilized to recognize nutrient-deficient pear leaves efficiently. The loss variation curve of the depth feature-based identification algorithms converges rapidly due to transfer learning, with the ViT-L/16 model converging fastest in terms of loss values and tending to a steady state after rapid convergence, but with a low identification accuracy of 78.68%. Although the ConvNeXt-Base model achieved the highest classification accuracy in the test set, the model converged with a more significant gap between the loss values of the training set and the validation set, indicating that the model was overfitting. Therefore, the single-feature model alone does not achieve an efficient diagnosis of nutrient-deficient pear leaves.

### 3.3. Pear Leaf Nutritional Deficiency Identification in Depth and Shallow Feature Fusion

This section analyzes the performance of pear leaf nutrient deficiency identification algorithms based on combining the three types of depth features extracted from different neural networks and three classification algorithms.

**Figure 11:**
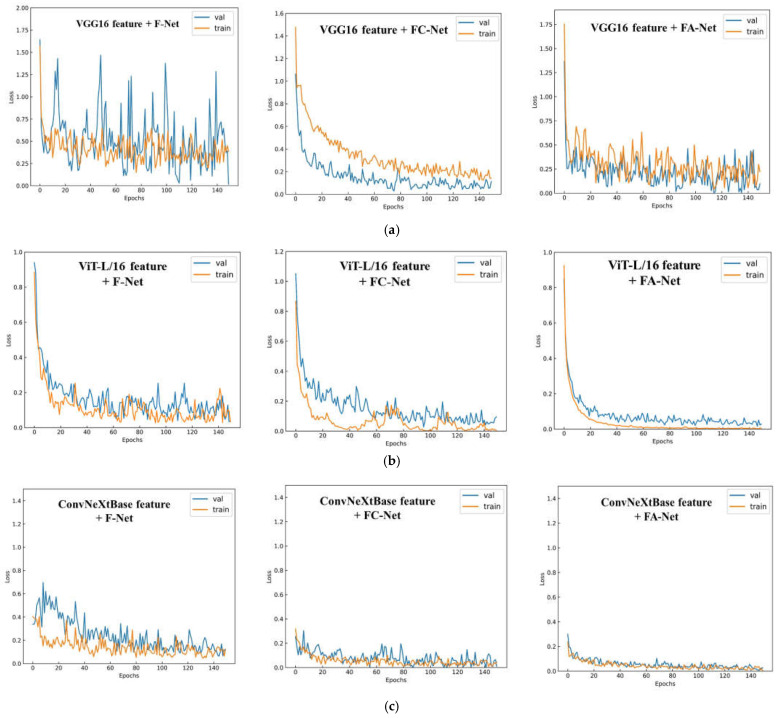
(**a**) Loss variation curve of the identification algorithm based on the VGG16 feature and shallow feature fusion. (**b**) Loss variation curve of the identification algorithm based on the ViT-L/16 feature and shallow feature fusion. (**c**) Loss variation curve of the identification algorithm based on the ConvNeXt-Base feature and shallow feature fusion.

As shown in Figure 11, the experimental results of the variation of loss values of the identification algorithm based on the fusion of shallow and deep features are presented. The identification algorithm based on the fused features has a faster fitting of the loss curve and a lower loss value when it is in a stable state after convergence than the identification algorithm based on the single feature. The identification algorithm based on the fusion of shallow and VGG16-depth features significantly fluctuates the loss value after convergence. The identification algorithm based on the fusion of ViT-L/16 depth and shallow features has a significant difference in the loss value between the training set and the test set. In contrast, the identification algorithm based on the fusion of ConvNeXt-Base depth and shallow features has the lowest loss value after rapid convergence, and after fast convergence, the lowest loss value is obtained, and the loss curves tend to be smooth.

Table 4 shows the accuracy, recall, precision, F1-score, and average precision (AP) of the pear leaf nutritional deficiency identification algorithm on the test set and its execution time per epoch. Training time is the average time consumed per epoch during the algorithm’s training. The precision, recall, and F1-score of the feature-fused identification algorithm are higher compared to the original model, and the training time of the feature-fused identification algorithm decreases significantly compared to the original model. The results show that the performance of the identification algorithm based on the fusion of depth and shallow features is better than that of the identification algorithm based on a single feature with the same classifier. The FA-Net as the classifier achieves the best classification performance, indicating that after the algorithm is improved, the network will consider the differences between positive and negative samples during training. Therefore, the identification algorithm based on shallow and depth feature fusion can more fully exploit the image features of deficient leaves to accurately identify pear leaf nutrient deficiency symptoms in complex environments.

In terms of time consumption in the test set, the recognition time of the feature-fused identification algorithm has increased compared to that of the original model due to the need for shallow feature extraction and feature fusion processes in each image recognition process, which increases the time of image recognition. However, the recognition time of each image is still controlled within 1 s, with the ability to be deployed in natural scenes. In the feature-fused identification algorithm, the training time of the algorithm on each batch is much lower than the testing time because, in the feature-fused algorithm, the number of extracted feature vectors is small. The training time in the neural network is very short, and the time consumption of the algorithm is mainly for extracting depth and shallow features. In the training process of 150 epochs, only the deep and shallow features are extracted at the first epoch and then sent to the neural network for training, so the average training time for each epoch is extremely short. During testing, the deep and shallow features are extracted again for each new image, increasing the image’s detection time.

In order to increase the comparison of correlation methods, this paper adds SVM and Random Forest as classifiers; the results are shown in Table 4, and relative to FA-Net, their results are worse. SVM and Random Forest use the grid search method to find the optimal parameters. The optimal parameter set of Random Forest is the maximum tree depth of 13, the minimum number of samples of leaf nodes is 20, the minimum number of samples required for internal node re-division is 120, and the maximum number of features is 7. SVM uses the RBF kernel function with a fixed radius parameter of *p* = 8 and a regularization parameter of 20.

**Figure 12:**
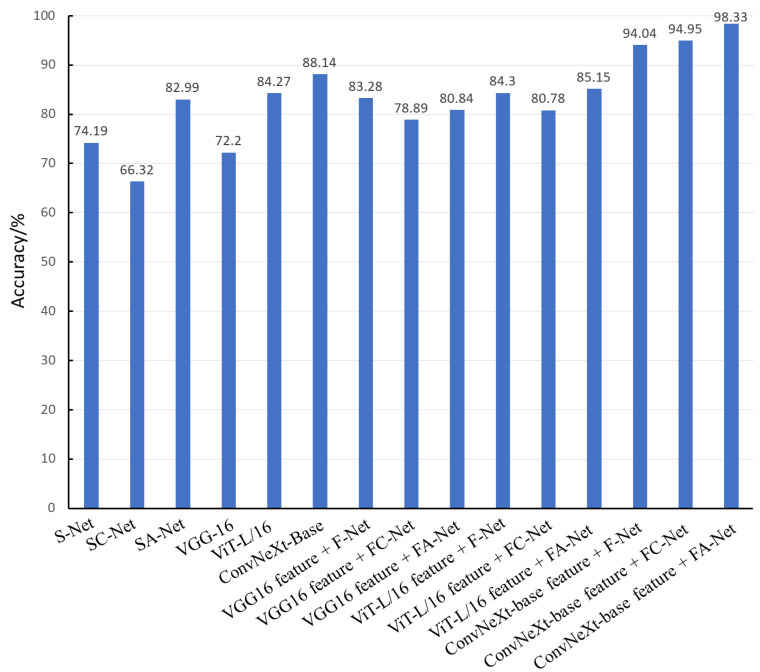
Comparison of the accuracy of pear leaf nutrient deficiency disease identification algorithms on the test set.

**Figure 13:**
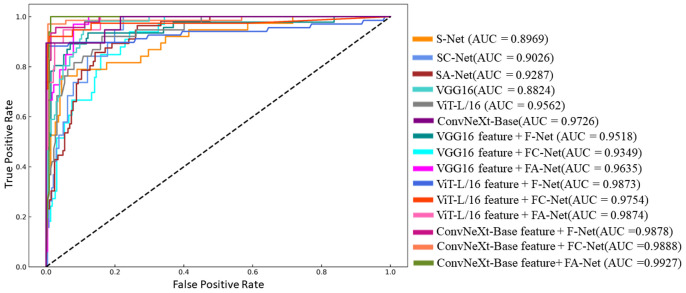
Receiver operating characteristic (ROC) curves of the pear nutrient deficiency disease identification algorithm.

Figure 12 shows the accuracy comparison of the 15 classification algorithms in this study on the test set. For the pear leaf nutrient deficiency identification task, the accuracy of the identification algorithm based on shallow features is lower than that of the identification algorithm based on depth features. Although the identification algorithm based on depth features has some advantages, it still cannot meet the high-precision classification needs of pear leaf nutrient deficiency in a targeted manner. From the characteristic details, both Fe-deficient and N-deficient leaves are yellowish, so it is more difficult for the identification algorithm based on depth to distinguish between them. The accuracy of the ConvNeXt-Base feature + FA-Net algorithm proposed in this study is 15.34 and 10.19 percentage points higher than that of the original ConvNeXt-Base model and the SA-Net algorithm, respectively. The algorithm can effectively capture the nuances between other nutritional deficiencies and performs well on the diagnosis task of pear leaf nutritional deficiency. As Figure 13 shows the ROC curve of the pear leaf nutritional deficiency identification algorithm used in this study, the AUC represents the area enclosed by the ROC curve and the coordinate axis. The larger the AUC area, the better the model’s performance.

**Figure 14:**
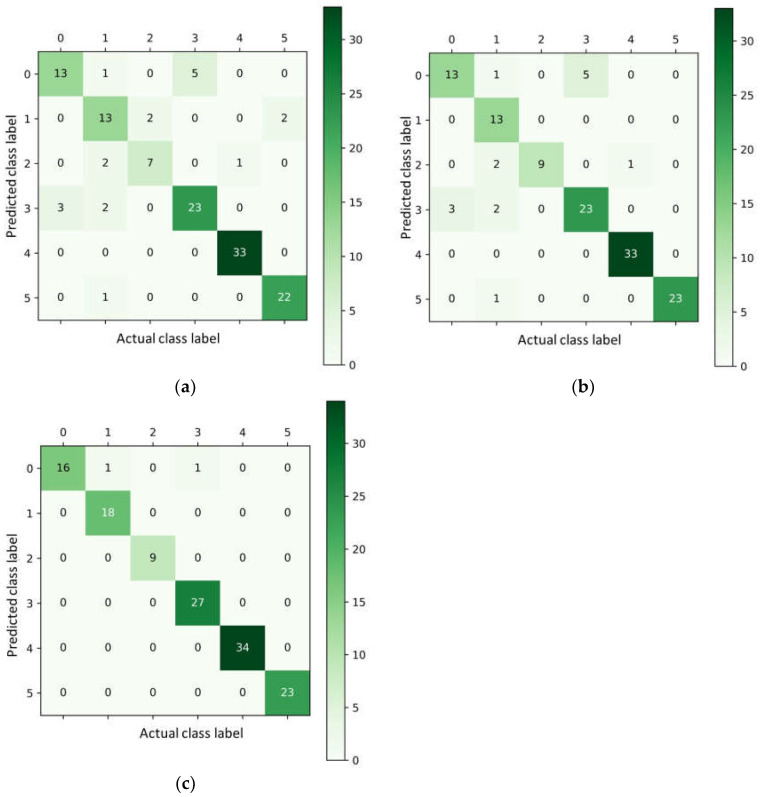
Model confusion matrix. Labels 0 to 5 represent six kinds of pear leaves, which are: 0 (Fe Deficiency), 1 (K Deficiency), 2 (Mg Deficiency), 3 (N Deficiency), 4 (P Deficiency), and 5 (Healthy). (**a**) Confusion matrix of FA-Net; (**b**) Confusion matrix of ConvNeXt-Base; and (**c**) Confusion matrix of ConvNeXt-Base feature + FA-Net.

The confusion matrix is one of the classification algorithm’s judging metrics. The row labels of the confusion matrix indicate the true category of the predicted image. The values at the row-column intersections indicate the number of corresponding column labels for which the category is predicted. The values at the diagonal indicate the number of correctly predicted labels, with larger values at the diagonal showing better model results. Figure 14a shows the confusion matrix based on the SA-Net algorithm, which has the best classification performance among the shallow feature-based identification algorithms, and Figure 14b shows the confusion matrix based on the ConvNeXt-Base model, which has the best classification performance among the depth feature-based identification algorithms. From the confusion matrix, the classification of P-deficient and healthy leaves is relatively good based on the extracted shallow features because the texture of healthy leaves is more apparent. The healthy leaves’ color is rich green, and the veins of the leaves will show purple-red when the leaves are deficient in P. P-deficient leaves and healthy leaves are better identified than other leaves. The leaves are primarily yellowish when they are deficient in Fe, Mg, and N, and the algorithm can easily confuse the three leaves and cause misclassification.

The ConvNeXt-Base network model showed a substantial decrease in the false identification rate for both leaves suffering from nutrient deficiency compared to the shallow feature-based identification algorithm. However, the false identification rate for Fe-deficient and N-deficient leaves with similar shapes and colors was still high. Figure 14c shows the confusion matrix of the ConvNeXt-Base feature + FA-Net algorithm. Compared with the SA-Net algorithm and the ConvNeXt-Base algorithm, the ConvNeXt-Base feature + FA-Net algorithm reduced the false identification rate of Fe-deficient and N-deficient leaves after fusing the shallow and depth features. The algorithm can more accurately distinguish the tiny differences between healthy and nutrient-deficient leaves, achieving clearer localization and more accurate identification of pear leaf nutrient deficiency symptoms. The confusion matrix of the ConvNeXt-Base feature + FA-Net algorithm exhibited the best performance and the highest average identification rate. In the case of fewer nutrient-deficient pear leaf samples, the fusion of depth and shallow features can more fully capture the differences between different nutrient deficiency features and construct a pear nutrient deficiency diagnosis method with high accuracy.

## 4. Conclusions

Diagnosing pear leaf nutrient deficiency using leaf visual images can guide formulation and fertilization early, inhibit the occurrence of nutrient deficiency, and prepare for a high yield and quality of pear fruit in the later stage. This study proposes a Fused Feature Multi-Head Attention Recoding Network (FA-Net) for diagnosing nutrient deficiency in pear leaf visual images by fusing shallow and depth features. Firstly, FA-Net achieved an average classification accuracy of 98.33% and an F1 score of 99.70% with the pear leaf nutrient deficiency dataset, outperforming either the shallow feature-based recognition algorithm or the deep network model using transfer learning. Secondly, it was found that different feature fusion methods had a significant impact on the classification performance of the identification model. The feature fusion method of FA-Net can achieve better classification accuracy based on ConvNeXt-Base depth features. Finally, the multi-head attention of FA-Net can fully exploit the connections between different vectors in the features, enabling the model to utilize the image features of nutrient-deficient leaves more thoroughly and efficiently. However, the algorithm proposed in this paper has some limitations because shallow features are not universal for different classification tasks. In conclusion, this manuscript explores a fusion feature method and the FA-Net algorithm for the nutrient deficiency of pear leaves that could improve the model’s performance. In future work, we plan to extend the method by using concepts of Super-Resolution [42] and Person Re-identification [43].

## Figures and Tables

**Table 1:** Diagnostic index of the nutrient content of pear leaves, which refers to the literature [32].

Diagnostic Index	Nutritional Deficiency	Suitable Nutrition
Iron deficiency/mg/kg	21~30	100
Potassium deficiency/%	<0.5	1.0~2.0
Magnesium deficiency/%	<0.06	0.25~0.80
Nitrogen deficiency/%	<1.3	2.0~2.4
Phosphorus deficiency/%	<0.09	0.12~0.25

**Table 2:** The number of samples in every category and the total number of samples in the training, validation, and test sets of the dataset.

Category	Training Samples	Validation Samples	Test Samples
Healthy	164	46	23
Iron Deficiency	118	34	16
Potassium Deficiency	134	38	19
Magnesium Deficiency	70	20	9
Nitrogen Deficiency	197	56	28
Phosphorus Deficiency	240	68	34
Total	923	262	129

**Table 3:** Accuracy of the pear nutrient deficiency identification algorithm.

Algorithm	Average Accuracy (%)
S-Net	74.19
SC-Net	66.32
SA-Net	84.60
VGG16	72.20
ViT-L/16	84.27
ConvNeXt-Base	86.00

**Table 4:** Accuracy, recall, precision, F1-score, and average precision (AP ) of the pear leaf nutritional deficiency identification algorithm on the test set and its execution time per epoch (training epoch contains 923 photos and test epoch contains 129 photos).

Algorithm	Recall (%)	Precision (%)	F1-Score (%)	AP (%)	Training Time (s)	Test Time (s)
S-Net	74.19	73.04	73.50	79.75	16.23	90.60
SC-Net	66.32	67.51	66.31	80.19	17.50	91.00
SA-Net	82.33	82.08	82.36	82.09	17.82	91.58
VGG16	67.07	70.62	70.95	82.79	59.25	25.32
ViT-L/16	84.82	85.67	85.23	85.90	64.70	27.63
ConvNeXt-Base	88.14	86.89	86.62	87.31	75.88	28.96
VGG16 feature + F-Net	84.30	82.24	82.79	82.81	22.23	108.20
VGG16 feature + FC-Net	80.71	82.47	81.36	82.79	23.50	109.30
VGG16 feature + FA-Net	84.87	85.69	85.76	85.06	23.82	110.72
ViT-L/16 feature + F-Net	79.20	78.88	78.65	87.84	22.73	108.71
ViT-L/16 feature + FC-Net	81.95	82.39	80.38	84.22	24.03	110.93
ViT-L/16 feature + FA-Net	83.98	83.30	86.82	84.39	24.32	111.21
ConvNeXt-Base feature + F-Net	94.06	94.57	94.21	95.70	23.33	110.54
ConvNeXt-Base feature + FC-Net	94.95	95.91	95.36	97.71	24.60	111.03
ConvNeXt-Base feature + FA-Net	98.52	98.14	99.70	98.83	24.92	111.41
ConvNeXt-Base fused feature + SVM	79.11	78.82	79.67	80.10	20.83	109.45
ConvNeXt-Base fused feature + Random Forest	80.23	81.12	79.95	80.13	23.21	110.36

## Data Availability

The datasets generated for this study are available on request from the corresponding author.
